# Association of Vitamin E with Rapid Thawing on Goat Semen

**DOI:** 10.1155/2014/964172

**Published:** 2014-05-11

**Authors:** Jurandy Mauro Penitente-Filho, Fabrício Albani Oliveira, Carolina Rodriguez Jimenez, Erly Carrascal, Júlio César Oliveira Dias, Gisele Dias Oliveira, Renata Gomes Silveira, Camila Oliveira Silveira, Ciro Alexandre Alves Torres

**Affiliations:** ^1^Department of Animal Science, Universidade Federal de Viçosa, 36570-900 Viçosa, MG, Brazil; ^2^Laboratory of Physiology and Animal Reproduction, Department of Animal Science, Universidade Federal de Viçosa, P. H. Rolfs Avenue, 36570-900 Viçosa, MG, Brazil; ^3^Department of Veterinary, Federal University of Viçosa, P. H. Rolfs Avenue, 36570-900 Viçosa, MG, Brazil; ^4^Federal University of Espírito Santo, University Campus, 29500-000 Alegre, ES, Brazil

## Abstract

The aim of this study was to evaluate the effects of vitamin E associated with rapid thawing on cryopreserved goat semen. Two bucks were used and eight ejaculates per animal were collected using artificial vagina. Semen was diluted with the following treatments: BIOXCELL (control), BIOXCELL + Equex (sodium lauryl sulphate) and BIOXCELL + vitamin E 100 **μ**M. Semen was packaged into 0.25 mL straws and cooled at 5°C for 1 hour. Freezing was performed in liquid nitrogen vapor (−155°C) during 15 minutes. Then, the straws were immersed in liquid nitrogen (−196°C). Straws were thawed at 38°C/60 seconds or at 60°C/7 seconds with immediate sperm analysis. Hypoosmotic swelling test was performed adding a 20 **μ**L aliquot of thawed semen to 1 mL of hypoosmotic solution (100 mOsm*·*Kg^−1^) followed by incubation during 60 minutes in water bath (38°C). Vitamin E did not affect any studied parameters (*P* > 0.05). Nevertheless, defrosting rate of 60°C/7 seconds improved sperm membrane functional integrity (*P* < 0.05). Current knowledge about goat semen cryopreservation is not sufficient to ensure high post-thawing recovery rates; thus, this study brings important data about using antioxidants and different thawing rates on cryopreservation process.

## 1. Introduction

Oxidative stress affects the organism when the generation of reactive oxygen species (ROS) exceeds the capacity of the cells to protect or repair themselves [1]. Reactive oxygen species can initiate lipid peroxidation and DNA damage leading to mutagenesis, carcinogenesis, and cell death, if the antioxidant system is impaired [2]. In physiological conditions, spermatozoa exhibit a capacity to generate ROS, but there is equilibrium between the generation of ROS and antioxidant strategies, leaving only a critical amount of ROS required for normal sperm functions, as capacitation, acrosome reaction, and fusion with the oocyte membrane [3]. Excessive production of ROS, however, results in peroxidation of the unsaturated fatty acids in sperm membrane, therefore resulting in defective sperm function [2].

In this context, the antioxidants appear as potentials constituent for cryoprotectors in the goat semen freezing process. Vitamin E is a primary component of sperm antioxidant system [4], and one of the main membrane protectors against ROS [5]. Cryopreservation of goat semen using Equex (sodium lauryl sulphate) showed beneficial effect on the parameters of the thawed goat semen at concentrations of 0.5 to 1% [6]. Therefore, Equex at a concentration of 0.008% v/v was used as an emulsifier of vitamin E [7, 8] in the freezing-thawing process.

The main damage caused by reheating occurs when the sperm passes through the critical zone between −50°C and −15°C or −5°C [9]. Moreover, sperm undergoes osmotic stress when duration of defrosting is insufficient to efflux the excess of cryoprotectants from within the cells. Sperm becomes turgid and smooth due to the abrupt dilution of the medium caused by defrosting of extracellular ice [10].

Thawing procedure should be performed at high temperature to minimize recrystallization. Thus, this study aimed to evaluate whether vitamin E associated with rapid thawing can reduce the damage on goat semen which is caused by freezing-thawing process.

## 2. Material and Methods

### 2.1. Animals: Collection and Physical Analyses of the Semen

Two bucks, Parda Alpina breed, from Universidade Federal de Viçosa, MG, with good body condition and three years old, were used. Animals were evaluated and approved for reproduction through andrological examination in agreement with quality patterns of fresh semen extolled by the Brazilian College of Animal Reproduction [11].

The collection period extended from April to May, 2010. Ejaculates were obtained through artificial vagina in the presence of an estrous doe restrained in collection trunk. Ejaculates were collected every 2 days, totalizing 8 ejaculates per animal.

After collections, semen physical analyses were performed: sperm motility (0–100%), vigor and mass movement (score from 0 to 5). Sperm morphology and concentration (sptz/mL) were done by separated samples. All the ejaculates that were used in this experiment followed the parameters summarized in Table 1 [11].

### 2.2. Stock Solutions of Vitamin E

An aliquot of Equex (sodium lauryl sulphate; 0.8 mL) was diluted in distilled water to prepare 100 mL of Equex solution (0.8% v/v), and 50 mL of this solution was distributed in 0.5 mL tubes and frozen at −20°C until use. Vitamin E (DL-*α*-Tocopherol acetate, Sigma-Aldrich) solution (10 mM) was prepared with the remaining 50 mL. This solution was homogenized with a magnetic stirrer, stored in 0.5 mL tubes, and frozen at −20°C. Vitamin E weight was done in a room with dim light due to its photosensitivity [7].

### 2.3. Semen Freezing and Experimental Design

After physical examination, 10 *μ*L of fresh semen was diluted (1 : 200) in Hancock solution [11] for sperm concentration analysis (haemocytometer method), for calculation of the final volume of diluent to be added.

For dilution, each ejaculate was divided into three treatments: BIOXCELL (control; Table 2), BIOXCELL + Equex, and BIOXCELL + vitamin E 100 *μ*M. Vitamin E and Equex were added to final dilutions and fixed in 10 *μ*L of stock solution per mL (1 : 100).

After dilution, semen was packaged into 0.25 mL straws with 50 × 10^6^ sperm. Straws were placed in a 20 mL test tube coated with refill (plastic bag) and placed into a 240 mL plastic container containing 125 mL of ethyl alcohol. Container was placed horizontally inside a refrigerator, with internal temperature at 5°C, with cooling rate of −0.38°C·min^−1^ for 45 minutes and 15 minutes of equilibration period [12].

Prefreezing was performed during 15 minutes in liquid nitrogen vapor with prefreezing rate of −10.7°C·min^−1^; for this, straws were placed 5 cm above liquid nitrogen [13]. After this period, straws were submerged in liquid nitrogen (−196°C) and stored in cryogenic cylinder.

Ten days after freezing, for each treatment, there were two types of thawing, both in water bath, as follows: at 38°C for 60 seconds or at 60°C for 7 seconds followed by 60 seconds at 38°C (Table 3). The semen was placed in tubes and homogenized for immediate analysis of sperm motility and vigor by phase contrast microscopy at 100x increase.

### 2.4. Hypoosmotic Swelling Test (HOST)

The functional integrity of the sperm membrane was evaluated by the hypoosmotic swelling test (HOST), using a hypoosmotic solution of 100 mOsmol·Kg^−1^. For the preparation of hypoosmotic solution, 9 g of fructose and 4.9 g of trisodium citrate were dissolved in 1000 mL of deionized water [14].

An aliquot of 20 *μ*L of thawed semen was added to 1 mL of hypoosmotic solution and incubated for 60 minutes in water bath at 38°C. Later, 0.5 mL of Hancock solution was added to the samples to fix them. Each HOST sample was mounted between glass slide and coverslip and examined in phase contrast microscope at 1000x increase. One hundred cells were analyzed per sample; the spermatozoa were classified by the presence or absence of coiled tail. Result was determined as percentage, and the calculation was done as follows: HOST% = (% change in the tail after HOST) − (% change in the tail before HOST).

### 2.5. Statistical Analysis

Experiment was carried out in a completely randomized design in factorial assay 3 × 2 (03 diluents × 02 thawing temperatures). For statistical analysis, the Statistical Analysis System [15] was used. Data of sperm motility and HOST were submitted to analysis of variance associated to Tukey's test using the PROC ANOVA, and interaction between diluents and thawing temperatures was assessed by the PROC GLM. Data of sperm vigor were analyzed by Wilcoxon test. Analysis of correlation between HOST and sperm motility and vigor was performed by Pearson's correlation and Spearman's correlation, respectively. A regression analysis of HOST and sperm motility was assessed by PROC REG. Significant level adopted was 5% of probability.

## 3. Results

Sperm motility, sperm vigor, and hypoosmotic swelling test showed no differences among studied diluents (*P* > 0.05; Table 4). Furthermore, there was no interaction between thawing temperature and diluents (*P* > 0.05).

Sperm motility and vigor showed no difference (*P* > 0.05) between the thawing rates (Table 5). Nevertheless, defrosting at 60°C/7 seconds increased functional integrity of sperm membrane (*P* < 0.05; Table 5).

## 4. Discussion

The effect of vitamin E on semen characteristics of domestic mammals has been extensively studied. Addition of vitamin E to animal diets showed goods results in sheep [16] and goats [17]. Nevertheless, inclusion of vitamin E to the semen extenders showed variable results among species. Vitamin E at a concentration of 100 *μ*M improved bull sperm membrane integrity, but it did not improve sperm motility and vigor [7]. Moreover, addition of vitamin E (100 *μ*M) to semen extender did not improve its quality in ram [18] and goats [8] supporting the present study.

The beneficial effect of Equex at concentrations of 0.5 to 1% on the parameters of the goat thawed semen has been reported [6]; nevertheless, these concentrations are larger than the 0.008% used in this study in which Equex was used as a Vitamin E emulsifier; however, even used as an emulsifier, Equex could improve bull sperm membrane integrity in the freezing process [7].

Thawing rate affects substantially quality of cryopreserved semen [19]. The ideal rate which recovers a great number of spermatozoa is variable among species. Thawing procedure should be performed at a high temperature to avoid recrystallization. The main damage caused by reheating occurs when the sperm passes through a critical zone between −50°C and −15°C or −5°C [9]. Sperm becomes turgid and smooth due to the abrupt dilution of the medium caused by defrosting of extracellular ice [10].

In this study, thawing rate of 60°C/7 seconds did not improve sperm motility when compared to thawing rate of 38°C/60 seconds (*P* > 0.05), probably because these two thawing rates do not possess sufficient differences in the formation of large ice crystals during crystallization to be able to affect goat sperm motility.

In bovine, there is a plateau in the relationship between thawing rate and sperm survival, so that increase in thawing temperature between 50°C and 70°C does not improve sperm motility [20]. It was hypothesized that, for goat semen, there is a lower plateau and temperatures between 37°C and 55°C also do not promote differences in the quality of thawed semen [21].

It is possible that temperatures higher than that used in this study could improve cryopreserved goat semen parameters. Tuli et al. [19] observed an increase on progressive motility and plasma membrane integrity of goat semen thawed at 70°C/7 seconds in comparison with the thawing rate of 37°C/2 minutes and 40°C/20 seconds. However, attention should be given to temperature and time of thawing; temperatures higher than 37°C leave the time as a critical variable. High temperatures can increase sperm mortality rates if applied improperly [22].

**Figure 1:**
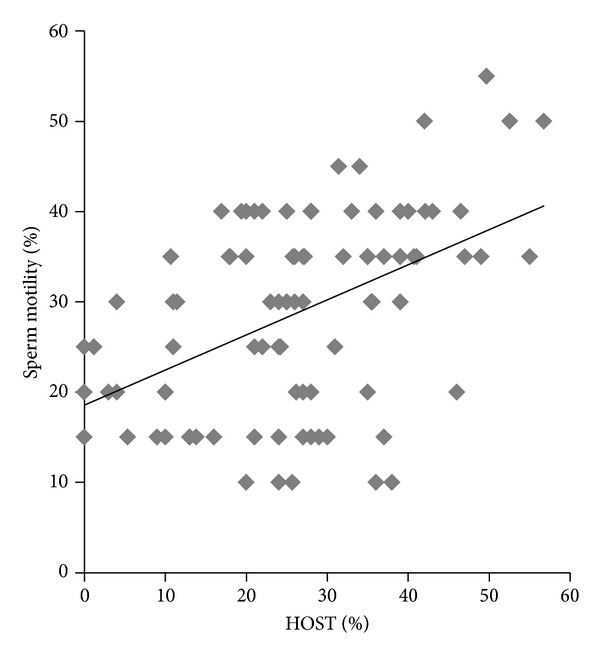
Dispersion graphic between sperm motility and HOST (*R* ^2^ = 0.22).

Nevertheless, the functional integrity of sperm membrane assessed by HOST was higher when semen was defrosted at 60°C/7 seconds (*P* < 0.05). This thawing rate probably is able to reduce formation of large ice crystals enough to protect the functional integrity of the goat sperm membrane. Correlation analysis showed no correlation between HOST and sperm vigor (*P* > 0.05), but there was correlation between HOST and sperm motility (*r* = 0.47; *P* < 0.001); besides, a regression analysis resulted in a linear function expressed by Sperm motility = 0.18547 + 0.38924 HOST (*P* < 0.001; *R* ^2^ = 0.22; Figure 1).

Although the HOST cannot be used as the unique parameter to assess sperm quality [23], it is a good supplemental test to improve the efficiency of the sperm analysis [24] which is supported by the *R* ^2^ value that was found in this study.

## 5. Conclusions

In conclusion, vitamin E did not affect the studied parameters of the thawed goat semen. Defrosting goat semen at 60°C/7 seconds followed by of 38°C/60 seconds can increase functional integrity of the goat sperm membrane but not the sperm motility and vigor. More studies are still required to understand the effective role of the antioxidants in goat semen as well as the effect of different thawing rates that may be used in the goat sperm freezing-thawing process.

## Figures and Tables

**Table 1:** Baseline values and characteristics of the ejaculates that were used in this study (mean ± standard error of mean).

Parameter	Baseline	Fresh semen
Progressive motility (%)	>70	84.1 ± 1.3
Sperm vigor (0–5)	>3	3.6 ± 0.1
Mass movement (0–5)	>3	3.2 ± 0.1
Total abnormal sperm (%)	<20	15.3 ± 1.8

**Table 2:** Chemical composition of the diluent (Bioxcell-IMV).

Components	g/L
Tris	2.3
Sodium citrate	6.2
Potassium chloride	0.8
Fructose	1.2
Monohydrate lactose	0.8
Glycine	0.2
Anhydrous glucose	0.5
Taurine	0.005
Gentamicin sulfate	0.24
Tylosin tartrate	0.33
Linco-Spectin 100	0.383
Glycerol	40.2
Hydrate of calcium lactate	0.7
Soy lecithin	1.5
Monohydrate citric acid	2.5
Ultrapure water	1000 mL

**Table 3:** Experimental design.

Semen samples	Treatment	Thawing
*n* = 16	Control	38°C/60 seconds
	Control	60°C/7 seconds followed by 38°C/60 seconds
	Equex	38°C/60 seconds
	Equex	60°C/7 seconds followed by 38°C/60 seconds
	Vitamin E 100 µM	38°C/60 seconds
	Vitamin E 100 µM	60°C/7 seconds followed by 38°C/60 seconds

**Table 4:** Sperm motility, sperm vigor, and hypoosmotic swelling test (HOST) of thawed goat semen cryopreserved with different media (mean ± standard error of mean).

Seminal parameters	Control	Equex	VE 100 *μ*M
Sperm motility (%)	30.2 ± 2.7	28.4 ± 2.8	28.3 ± 2.7
HOST (%)	25.5 ± 4.0	27.4 ± 3.9	26.4 ± 3.0
Sperm Vigor	2.0 ± 0.1	1.9 ± 0.1	2.0 ± 0.1

*P* > 0.05; VE: vitamin E; HOST: hypoosmotic swelling test.

**Table 5:** Sperm motility, sperm vigor, and hypoosmotic swelling test (HOST) of thawed goat semen thawed at different temperatures (mean ± standard error of mean).

Seminal parameters	38°C/60 s	60°C/7 s
Sperm motility (%)	27.2 ± 1.5^a^	30.7 ± 1.6^a^
HOST (%)	23.8 ± 1.9^b^	29.3 ± 2.0^a^
Sperm vigor	1.9 ± 0.1^a^	2.1 ± 0.1^a^

^a,b^Different letters in the row are statistically different (*P* < 0.05); HOST: hypoosmotic swelling test.
